# Coupling Fault Diagnosis Based on Dynamic Vertex Interpretable Graph Neural Network

**DOI:** 10.3390/s24134356

**Published:** 2024-07-04

**Authors:** Shenglong Wang, Bo Jing, Jinxin Pan, Xiangzhen Meng, Yifeng Huang, Xiaoxuan Jiao

**Affiliations:** Aeronautics Engineering College, Air Force Engineering University, Xi’an 710038, China; phm_wsl@outlook.com (S.W.); jingbo_sensors@outlook.com (B.J.); panjinxin_sensor@outlook.com (J.P.); mengxz_sensor@outlook.com (X.M.); huangyiff@189.cn (Y.H.)

**Keywords:** coupling fault diagnosis, graph neural networks, interpretability, dynamic vertex

## Abstract

Mechanical equipment is composed of several parts, and the interaction between parts exists throughout the whole life cycle, leading to the widespread phenomenon of fault coupling. The diagnosis of independent faults cannot meet the requirements of the health management of mechanical equipment under actual working conditions. In this paper, the dynamic vertex interpretable graph neural network (DIGNN) is proposed to solve the problem of coupling fault diagnosis, in which dynamic vertices are defined in the data topology. First, in the date preprocessing phase, wavelet transform is utilized to make input features interpretable and reduce the uncertainty of model training. In the fault topology, edge connections are made between nodes according to the fault coupling information, and edge connections are established between dynamic nodes and all other nodes. Second the data topology with dynamic vertices is used in the training phase and in the testing phase, the time series data are only fed into dynamic vertices for classification and analysis, which makes it possible to realize coupling fault diagnosis in an industrial production environment. The features extracted in different layers of DIGNN interpret how the model works. The method proposed in this paper can realize the accurate diagnosis of independent faults in the dataset with an accuracy of 100%, and can effectively judge the coupling mode of coupling faults with a comprehensive accuracy of 88.3%.

## 1. Introduction

A mechanical structure is affected by temperature, vibration, humidity, electromagnetic, shock, and other external stresses during operation. The external stresses act on a large number of components at the same time and affect their reliability synchronously [1]. Meanwhile, during the operation of the equipment, there are internal stresses such as friction and collision between the equipment [2], and their reliability is influenced by each other. Under the same working conditions, there are widespread cases of multi-fault coupling derivation in mechanical equipment [3,4]; furthermore, the coupling of faults may aggravate the derivation speed of faults. Therefore, the accurate identification of coupling faults can diagnose and warn of the occurrence of faults in advance, providing an important reference for maintenance decisions, which has an important application value [5].

Recently, scholars have made important contributions to the research on coupling the fault diagnosis of rotating machinery. Some scholars have studied the frequency domain characteristics of vibration signals and classified multiple faults based on the frequency domain characteristics. In coupling fault diagnosis, fault classification is usually carried out by decoupling, and bearing fault features are extracted. Mishra et al. collected bearing vibration signals under uncertain speed conditions and proposed the STFNet network to extract vibration features and classify faults at different rotating speeds [6]. Tao et al. proposed a cross-domain diagnosis method based on time–frequency domain information fusion, which uses the joint distribution distance to cluster fault features in the time–frequency domain [7]. Zhang et al. selected three indices insensitive to defect position as feature vectors for bearing fault diagnosis, and used the KNN algorithm for fault classification [8]. Dezun et al. proposed iterative generalized demodulation (IGD) to extract the feature frequency of instantaneous faults and carried out feature extraction and fault diagnosis for bearing faults with multiple faults and variable rotating speed [9]. Neisi et al. used UKF to estimate the vibration characteristics of rotating machinery to achieve data fitting and fault identification under multi-fault conditions [10]. Wang et al. selected the data channel by the Pearson correlation coefficient combined with the GRU method for multiple fault diagnosis, and time judged the severity of the fault [11].

Some other scholars have used the neural network data-driven method to classify the multiple faults of rotating machinery. By using the self-consistency of the convergence model [12], the neural network arranges the chaotic model parameters through gradient descent to make them show functional consistency on a macro level, and is widely used in the research of the fault diagnosis of mechanical equipment. However, the black box attribute of the neural network brings difficulties to the interpretation of the model. Although the neural network has a strong performance, it reduces the reliability of the model, which has a negative impact on the engineering application of the neural network to some extent. In the study of the fault diagnosis of rotating machinery, Zhang et al. diagnosed bearing multiple faults by improving the CNN network pool layer and fully connected layer structure [13]. Gong et al. used the finite element simulation method to expand the non-equilibrium data of bearing multiple faults, and the transfer learning method was used to diagnose the faults [14]. Deng et al. proposed the MgNet model to diagnose multi-bearing faults at the system level by collecting vibration signals of the auxiliary bearings [15]. Shi et al. proposed the WDCNN method to classify bearing faults by introducing the multi-scale large convolutional kernel and GRU network, and divided the bearing life cycle into the health stage and fault stage [16]. Hadi et al. determined the structure of the fault diagnosis neural network through reinforcement learning and other methods, which further veiled the explainability of the model [17].

On the cutting edge of machine learning, the generative large model is widely used to manufacture and output knowledge, but its reliability is difficult in terms of meeting the high accuracy of fault diagnosis and other engineering fields [18]. In order to improve the reliability of the model, scholars have carried out research on the interpretability of the model. In the study of interpretable neural networks, Zhang et al. [19] classified interpretable models according to three dimensions: type of engagement (passive and active interpretation methods), the type of explanation, and the focus (from local to global explainability). Li et al. used the U-Net network and integrated signal segmentation technology to mine interpretable fault characteristic information to realize fault diagnosis [20]. Che et al. used the multi-domain element transfer learning framework based on deep convolutional neural networks to reverse reconstruct the fault classification results through the fusion of hierarchical class activation mapping to obtain the mapping relationship between the input and output, which is a global ante hoc interpretable model with feature decomposition [21]. The DIGNN model proposed in this paper is a global ante hoc interpretable model with rule extraction.

The graph neural network was proposed in 2009 [22]. Due to its unique network topology, it has the ability to represent graph data and analyze related data. Currently, it is mainly used in natural language processing [23,24], traffic flow analysis [25,26], molecular structure modeling [27,28], and other fields. According to its structure, it can be divided into two types: spatial-based and spectral. The spectral-based method uses the graph convolution filter to denoise the node data and extract the main features of the signal. Michael et al. [29] extended CNN to the field of graph signal processing, and established convolution check data based on spectrum to carry out convolution operations, reducing its operational complexity. Defferrard et al. [30] improved the efficiency of graph coarsedness when CNN was generalized to GNN by establishing the operation of balanced binary tree record graph coarsedness. The spatial based method is used to aggregate information of the node neighborhood. Gama et al. [31] established the input time series by means of graph aggregation to implement CNN transplantation for unstructured graph data.

Li et al. [32] first applied the graph neural network to fault diagnosis research and established relevant research benchmarks. This method converted time series single-channel data into graph structure to respectively realize fault diagnosis of rotating machinery. However, the topology structure of the graph data constructed by this method lacks physical meaning and has poor interpretability. Gao et al. used GNN to conduct one-shot model training and diagnosis for bearing fault types under varying working conditions [33]. Man et al. [34] took the data collected by multiple sensors as the nodes in the figure respectively, and used GAT to diagnose the fault of the train steering gear. Zhang et al. proposed a graph neural network (GNN) method based on the Granger causality test, which decomposed vibration signals into noise signals and fault signals. The graph data were established for nodes based on fault types to classify faults [35]. The methods mentioned above fix the topology structure of graph data, which is difficult to adapt to the uncertain topology structure caused by the unknown fault type input of real-time data in engineering applications. The methods mentioned run in black box mode, but they still fully embody the excellent performance of the graph neural network in the field of fault diagnosis. In this paper, an interpretable DIGNN method was proposed based on the graph neural network, which can effectively utilize the characteristics of node correlation to realize the coupling fault diagnosis of rotating machinery. The main contributions of this paper are as follows:

(a) A topology construction method of dynamic vertex data for graph neural networks is proposed, which is suitable for topology-based correlation analysis.
(b) An explainable coupling fault diagnosis method is proposed, which gives physical meaning to the data-driven method based on the graph neural network.
(c) In this paper, the bearing coupling fault was analyzed as an example, and the test results show that the method can realize coupling component analysis on the basis of coupling fault diagnosis.

The rest of this paper is organized as follows. Section 2 describes an interpretable DIGNN method based on the graph neural network with the characteristic of node correlation for coupling fault diagnosis. In Section 3, the overall process of the DIGNN algorithm is described, and the pre-processing scheme of wavelet transform and the network structure of DIGNN are specifically described, and the interpretability of the model is analyzed. In Section 4, the dataset used in this paper is first introduced in Section 4.1, then the data are pre-processed using wavelet transform, and the graph data containing the fault topology are constructed in Section 4.2. Then, the method proposed in this paper is used to diagnose the rotating mechanical coupling fault compared with other benchmark methods in Section 4.3. The classification of model coupling faults is explained and analyzed. Finally, conclusions are drawn in Section 5.

## 2. Interpretability of Graph Neural Networks

In the fault diagnosis of independent fault modes, only the feature extraction of the single fault mode needs to be considered. While under the condition of multiple fault coupling, there are correlation features between coupled fault modes, and the traditional CNN network cannot extract the implicit correlation between multiple faults. Graph neural networks are able to propagate relevant information between nodes through edges, thus providing interpretability to coupled fault diagnosis. Some notations used in this paper are shown in Table 1:

In graph 𝒢=(𝒱,ε), 𝒱 represents the vertices in the graph; ε is the edge connecting the vertices; $\varepsilon =\left\{e_{ij}|x_{i},x_{j}\in 𝒱\right\},$ $|\varepsilon |\leq N^{2}$. Let A represent the symmetric adjacency matrix, D denote the degree matrix, and $X\in R^{N\times d}$ be the input data. N and d are the number of nodes and the feature dimension, respectively. L=D−A is a symmetric matrix Laplacian matrix, and the Fourier basis U is obtained by the spectral decomposition of L.

(1) $$L=U\Lambda U^{-1}=Udiag[\lambda_{1},\ldots ,\lambda_{N}]U^{-1}$$

The matrix obtained by spectral decomposition is orthogonal, $UU^{T}=E$, $L=U\Lambda U^{T}$, the Fourier transform of x is $\hat{x}=U^{T}x$, and its inverse transformation is $x=U\hat{x}$, the continuous orthogonal basis on the graph is converted to the basis of the Fourier transform for graph convolution operation:

(2) $$\begin{array}{cc} x{*}_{𝒢}g & =U((U^{T}g)⊙(U^{T}x))\\ & =Ug_{\theta }U^{T}x \end{array}$$

where $g_{\theta }=U^{T}g=g_{\theta }(\Lambda )$ denotes the diagonal matrix and ⊙ is the Hadamard product. The Chebyshev polynomial $T_{k}(x)=2xT_{k-1}(x)-T_{k-2}(x)$, $T_{1}(x)=x$, $T_{0}(x)=1$ is introduced into the inner product of the vector. Then, the ChebyNet expression is obtained, and the nodes in the K−hop neighborhood are aggregated as follows:

(3) $$x{*}_{𝒢}g\approx \sum_{k=0}^{K-1}\theta_{k}T_{k}(\tilde{L})x$$

where $\tilde{L}=2/\lambda_{\max}(L-I_{N})$ is the standardized Laplacian eigenvalue and $\lambda_{\max}$ is the largest eigenvalue. Take K=2 to simplify ChebyNet further:

(4) $$\begin{array}{cc} x{*}_{𝒢}g & \approx \theta_{0}x+\theta_{1}\left(L-I_{N}\right)x\\ & =\theta_{0}x-\theta_{1}D^{-\frac{1}{2}}AD^{-\frac{1}{2}}x \end{array}$$

Defining $\theta =\theta_{0}=-\theta_{1}$, then $x{*}_{𝒢}g\approx \theta \left(I_{N}+D^{-\frac{1}{2}}AD^{-\frac{1}{2}}\right)x$. Let ${\tilde{D}}_{ii}=\sum_{j}{\tilde{A}}_{ij}\tilde{A}=A+I_{N}$ and ${\tilde{D}}^{-\frac{1}{2}}A{\tilde{D}}^{-\frac{1}{2}}=I_{N}+D^{-\frac{1}{2}}AD^{-\frac{1}{2}}$, so the GCN interlayer formula [36] is obtained as follows:

(5) $$X^{l+1}=\sigma \left({\tilde{D}}^{-\frac{1}{2}}A{\tilde{D}}^{-\frac{1}{2}}X^{l}W^{l}\right)$$

where σ is the nonlinear mapping function, $W^{l}$ is the weight matrix of the lth layer, and $X^{l}$ is the input variable of the lth layer ($X^{0}=X$). $\tilde{A}=A+I_{N}$ is the adjacency matrix of adding node self-information, and the aggregation of node information in the node-hop neighborhood is realized by symmetric normalization of the adjacency matrix $D^{-\frac{1}{2}}\tilde{A}D^{-\frac{1}{2}}$. Through k layers of the GCN network, the information of vertices within k−hop are aggregated. The symmetric standardization defines the direction of information transmission between nodes, which not only avoids the difference in data measurement scale caused by the difference in node degree, but also considers the amount of information of the two nodes connected by the edge [37].

**Figure 1:**
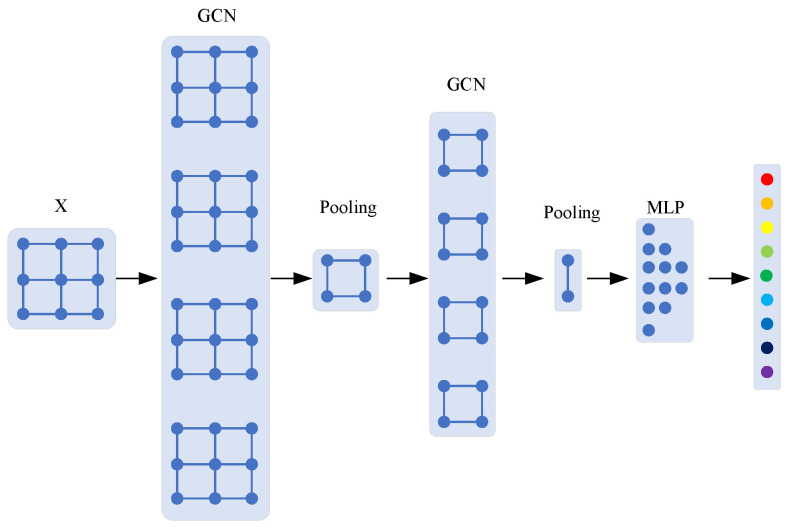
Flowchart of a traditional GCN algorithm.

The traditional GCN algorithm uses a data-driven approach to build data topology, which can be modified when information is transferred between layers, as shown in Figure 1.

## 3. Algorithm Flow

### 3.1. Data Preprocessing

**Figure 2:**
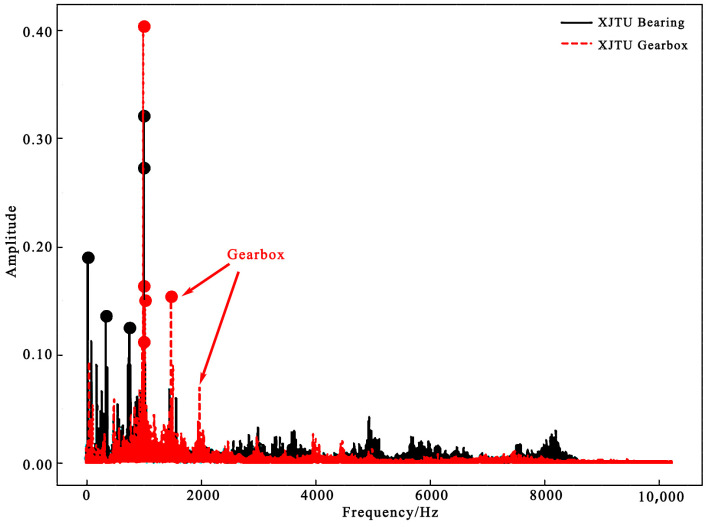
Vibration signal spectrum of XJTU Bearing datasets and XJTU Gearbox datasets.

In this paper, the coupling fault was indicated by a node in the graph, and the coupling topology was constructed by connecting the coupling fault node with a single fault node. In order to provide the ability of fault diagnosis analysis and diagnosis of new data, this paper took the fault to be classified as a node in the graph, which is called a dynamic vertex, and constructed the edge vector between the node and the other nodes. In actual condition, bearings and gear structures often work at the same time, and their vibration signals affect each other, as illustrated in Figure 2. Comparing the bearing data doped with gear signals in the XJTU Gearbox dataset with the pure bearing vibration signals in the XJTU-SY Bearing datasets, the rotation of gears will generate high-frequency signals, which will affect the simplicity of the bearing characteristics. Therefore, this paper also took the gear faults as nodes to provide negative samples for the fault diagrams and reduce the impact of gear faults on the fault diagnosis results [38].

For rotating machinery, the vibration signal characteristics are mainly determined by the vibration direction, rotation frequency, and vibration amplitude. In order to improve the interpretability of the method and reduce the uncertainty of the data-driven training of graph neural networks, this paper adopted wavelet transform to preprocess the data [39]. Morlet wavelet basis was used to transform the data, and the bandpass filter ensured that it had good time–frequency domain localization characteristics and could accurately locate the frequency range. The expression is as follows:

(6) $$\psi (t)=\exp(i\omega_{0}t)\exp(-\frac{t^{2}}{2})$$

By expanding and translating at different scales a and amounts of displacement b, the wavelet family is obtained:

(7) $$\psi_{a,b}(t)={\left|a\right|}^{\frac{1}{2}}\psi \left(\frac{t-b}{a}\right)=\exp(\frac{i\omega_{0}(t-b)}{a})\exp(-\frac{{\left(t-b\right)}^{2}}{2a^{2}})$$

where $a_{i}=2f_{\psi (t)}*totalscale/i$, in which $f_{\psi (t)}$ is the center frequency of the wavelet ψ(t) and totalscale is the number of scales. After, the input signal x is transformed into a two-dimensional signal (t, $a_{i}$, value), in which value=abs(WT(a,b)). The formula of continuous wavelet transform is:

(8) $$\begin{array}{cc} WT(a,b) & =\int_{R}x(t)\psi_{a,b}(t)dt\\ & =\int_{R}x(t){\left|a\right|}^{\frac{1}{2}}\psi \left(\frac{t-b}{a}\right)dt \end{array}$$

The data topology is constructed according to the prior knowledge, in which the fixed vertices are the standard features of the wavelet transform of each type of fault, the dynamic vertices are the data to be measured, and the graph data X is formed together.

### 3.2. Coupling Fault Diagnosis

The data were divided into the training set and the test set, and the partition ratio was 8:2. In this paper, the cross-entropy error was used as the error calculation method; the label y_lable∈R was converted into one-hot code $y\in R^{N}$ and used to calculate the cross-entropy with the model output $\hat{y}\in R^{N}$:

(9) $$C(y,\hat{y})=-\frac{1}{n}\sum_{x}\left[y\ln\hat{y}+(1-y)\ln(1-\hat{y})\right]$$

The loss function consists of two parts, Loss1 and Loss2, where Loss1 is the classification error of the fault type to be diagnosed and Loss2 is the classification error of the fault type of other nodes. The total loss function is as follows.

(10) $$\begin{array}{cc} Loss & =Loss1+\alpha Loss2\\ & =C(y_{N},{\hat{y}}_{N})+\alpha \sum_{i=1}^{N-1}C(y_{i},{\hat{y}}_{i}) \end{array}$$

Since the model focuses on the fault classification of unknown faults, we set α<1 so that the accuracy of node classification was ensured first in this paper.

### 3.3. Algorithm Flow

**Figure 3:**
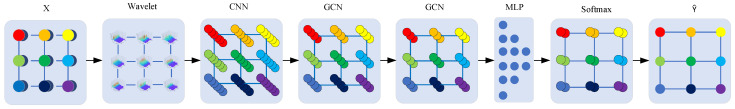
Flowchart of the DIGNN algorithm with dynamic vertex. Each color represents a fault type.

The algorithm flow of the algorithm proposed in this paper is shown in Figure 3.

(a) The input data $X^{0}=X=(x_{1},x_{2},\ldots ,x_{N})$, $X^{0}\in R^{N\times d}$ are composed of node features $x_{n}$$\in R^{d}$ of N types of faults, where d is the feature dimension. The Nth node is the dynamic vertex (i.e., the fault node to be diagnosed).
(b) After $X^{0}$ is transformed by wavelet, $X^{1}=Wavelet(X^{0})$, $X^{1}\in R^{N\times f\times S}$ where f is the number of the frequency spectrum and S is the size of the wavelet scale. The wavelet transform raises the dimension of one-dimensional data, endows the data with more intuitive features, and at the same time, carries out data preprocessing, which is conducive to the subsequent feature extraction of the neural network.
(c) After the signal is convolved on the two-dimensional spectrum data, the number of convolution nuclei is f, and $X^{2}\in R^{N\times f}$ is obtained. Through model training, it extracts the feature assignment in $X^{2}$ at each frequency and carries out standardization processing. In the process of CNN, only independent convolution processing is carried out for each node, that is, no data information from other nodes is involved.
(d) Through the two-layer GCN network, it can be obtained that $X^{3}=GCN^{1}(X^{2})$, $X^{4}=GCN^{2}(X^{3})$, where $X^{3}\in R^{N\times \frac{f}{2}}$, $X^{4}\in R^{N\times \frac{f}{4}}$. The feature extraction of high-frequency and low-frequency features is further carried out in the way of dichotomy, which is similar to the wavelet packet decomposition technology [40]. At the same time, the specific features of each type of fault are extracted through model training.
(e) Through the fully connected MLP, there is further dimensionality reduction of the data, $X^{5}=MLP(X^{4})$, $X^{5}\in R^{N\times \frac{f}{8}}$. Finally, fault classification $\hat{Y}$ is output through the Softmax layer.
(f) In the process of model training, the output value ${\hat{y}}_{N}$ of the last layer of node $x_{N}$ is used as the training label to optimize the model parameters. After the model is established, node $x_{N}$ will play the same role with other nodes in the model operation, which only carry out independent output in the model output phase.

### 3.4. Interpretability Analysis

Explainability is defined as the ability to explain to people in plain language. Graph neural network itself has distinct physical significance because of the topology of its nodes. The interpretability of the structure is made clear by assigning the input data with relation to the node. The DIGNN model proposed in this paper explores and optimizes the interpretability of the model in the specific application of coupling fault diagnosis, so this model is an interpretable model [41].

(a) Each node in 𝒢 is a type of fault data, and the topological structure maintains the input structure from beginning to end. Each node has a clear physical meaning, and each node in the output data $\hat{Y}$ corresponds to the classification of various types of fault data.
(b) The vibration signal is converted into the time–frequency domain signal by wavelet transform, which provides the data with a clear physical meaning. Due to the introduction of data topology, the physical meaning of each vertex data $X^{1},X^{2},X^{3},X^{4},X^{5}$ remains stable, even if the data dimension is changed under the condition that the GCN network structure remains unchanged, and all of them are linear transformations of the vibration amplitude of this type of fault at a specific frequency.
(c) The similar characteristics of similar faults in coupling faults are enhanced by aggregation operation:
  (11) $$x_{n}=aggregation(N\{x_{n}\})$$
  where $N\left\{x_{i}\right\}=\left\{x_{j}|e_{ij}\in \varepsilon \right\}$, and the fault characteristic information of related nodes is aggregated through the target vertex of GCN. The nodes at both ends are comprehensively considered by the symmetric standardization operation $D^{-\frac{1}{2}}\tilde{A}D^{-\frac{1}{2}}$. The degree of coupling fault is relatively large because it is related to multiple fault nodes, and the symmetric standardization can reduce the weight of such nodes in the aggregation, effectively avoiding the influence of unrelated fault type data on the aggregation of adjacent nodes.

## 4. Dataset Introduction and Data Processing

### 4.1. Dataset Introduction

**Figure 4:**
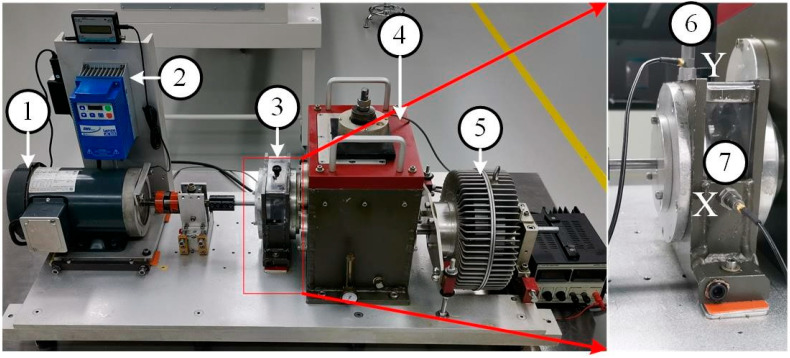
Fault injection experiment platform. 1. Motor, 2. Controller, 3. Bearing, 4. Gearbox, 5. Loading, 6. Vertical accelerometer, 7. Horizontal accelerometer.

**Figure 5:**
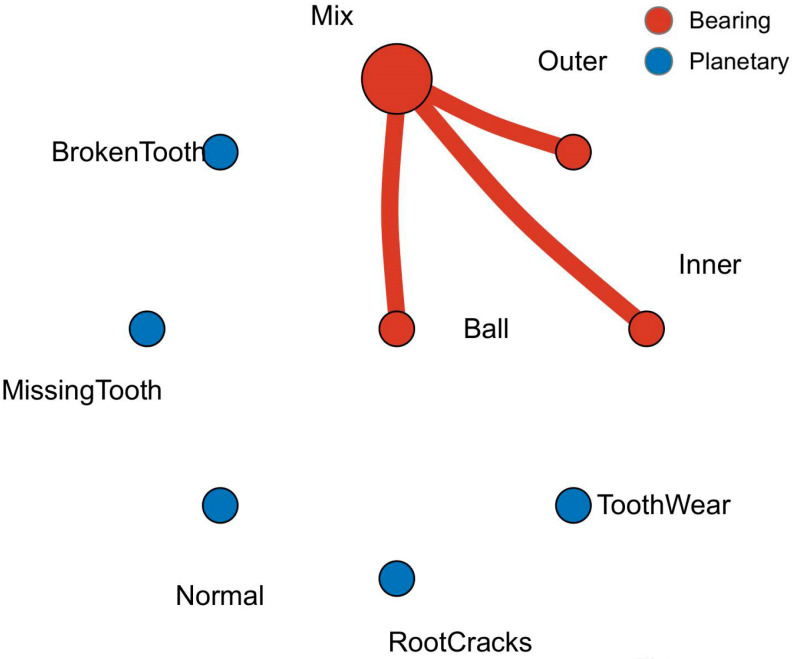
Topology of the coupling faults.

In this paper, the data in the XJTU Gearbox dataset were used. Four planetary gear failure modes and four bearing failure modes were injected in the experiment. As shown in Figure 4, injected gear failures included tooth wear, missing tooth, root cracks, and broken tooth. The injected bearing faults included ball bearing faults, inner race faults, outer race faults, and coupling faults of the above three bearing faults. Together with the normal state, a total of nine states of the type vibration signals were collected. The fault relationship topology is shown in Figure 5.

In the experiment, transverse and longitudinal vibration sensors were installed to collect the state data. In this paper, radial and vertical vibration signals were selected for processing, and the sampling frequency was 20.48 kHz

### 4.2. Data Preprocessing

**Figure 6:**
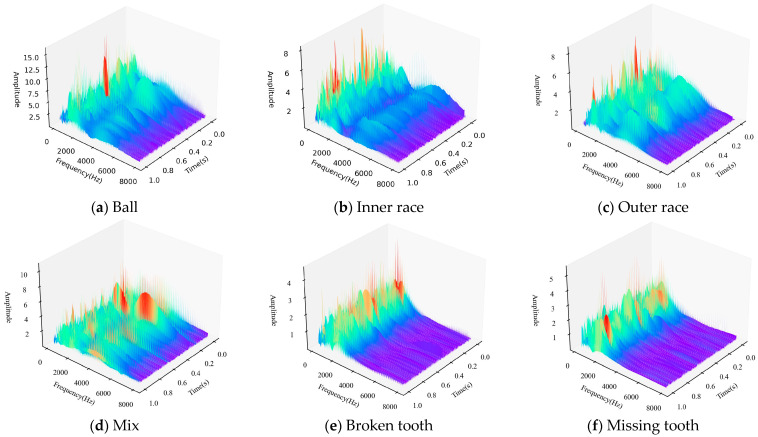

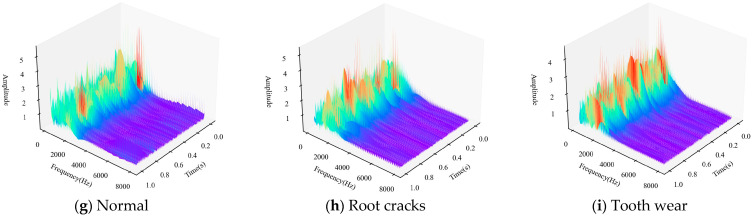
Time–frequency feature extraction of the fault data.

The data were preprocessed by wavelet transform before input into the neural network. As shown in Figure 6, the signals in the time–frequency domain of the bearings and gears were mainly concentrated within 8000 Hz, so the time–frequency domain signals in the frequency band from 0 to 8000 Hz were selected in this paper, and the time–frequency domain signals of various faults are shown in Figure 6.

It can be seen from Figure 6 that fault coupling is not a simple feature superposition, but contains a complex physical mechanism. In this paper, the data-driven graph neural network method was used to simulate the physical mechanism and achieve multi-fault coupling analysis.

**Figure 7:**
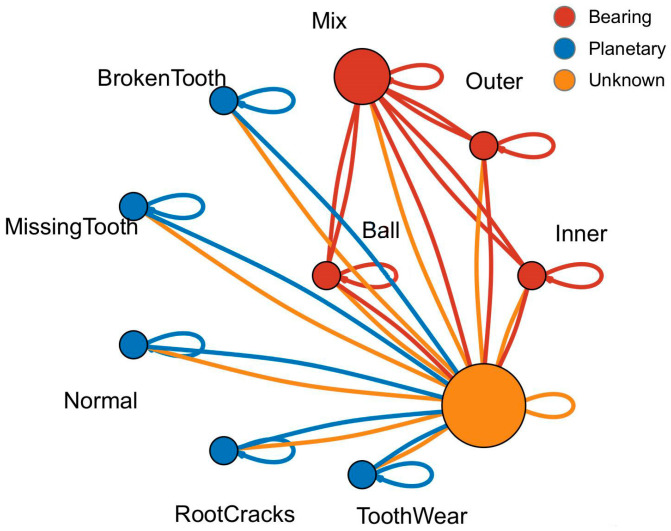
Topology and relationship of the coupling fault data with self-loops.

After analyzing the fault types, the fault data topology was established according to the coupling relationship between faults. The graph structure established in this paper was an undirected graph containing self-loops, as shown in Figure 7. The data topology was composed of 10 nodes $𝒱=\{x_{1},x_{2},\ldots ,x_{10}\}$. Fault data were randomly selected in the dataset and filled with flexible nodes to form a training set, corresponding to the orange node in the figure, $x_{4}$ is the coupled fault data, and the other nodes are independent fault types. The data topology of the graph consisted of 34 edges $e_{i,j}\in \varepsilon$, and the dynamic vertex was assumed to be related to all vertices because of its type and the unknown correlation with other vertices (i.e., $e_{1,10},e_{9,10},\ldots ,e_{10,10}=1$).

In the training set data topology, the data X except the dynamic vertex $x_{10}$ was randomly sampled in the data sample corresponding to the fault type. In order to reduce the difficulty of test set creation and application, this paper took the average value of all kinds of known fault data after wavelet transform as the fixed vertices of the test set: $\left\{x_{1},x_{2},\ldots ,x_{9}\right\}$.

**Figure 8:**
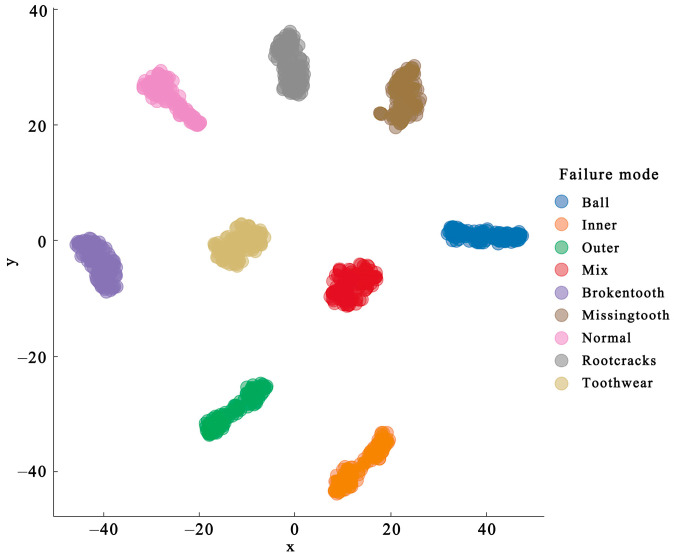
Fixed vertex feature in feature space.

After wavelet transform, the data of each fault type were clearly divided in the data space, as shown in Figure 8, which helped to reduce the influence of uncertainty brought by the graph neural network. In the fault space, the distance between the coupling fault and its fault type was relatively close, reflecting the coupling effect between faults. The obvious boundary between the bearing and gear faults was obtained only by wavelet transform.

### 4.3. Coupling Fault Diagnosis

First, the model proposed in this paper was used for coupling fault diagnosis of the data. The training set contained 540 samples, the validation set contained 180 samples, and the test set contained 180 samples. In each sample, which can be called a graph cluster, $x_{10}$ was selected randomly from nine fault types. The model adopted the SGD optimization method, the learning rate was 0.01, and the momentum was defined as 0.9. A convolutional neural network with a 100 × 1024 × 1024 filter was used in series with two layers of GCN networks. The filter size in the first layer of GCN was 1024 × 512 and the second GCN layer contained a 512 × 256 filter.

**Figure 9:**
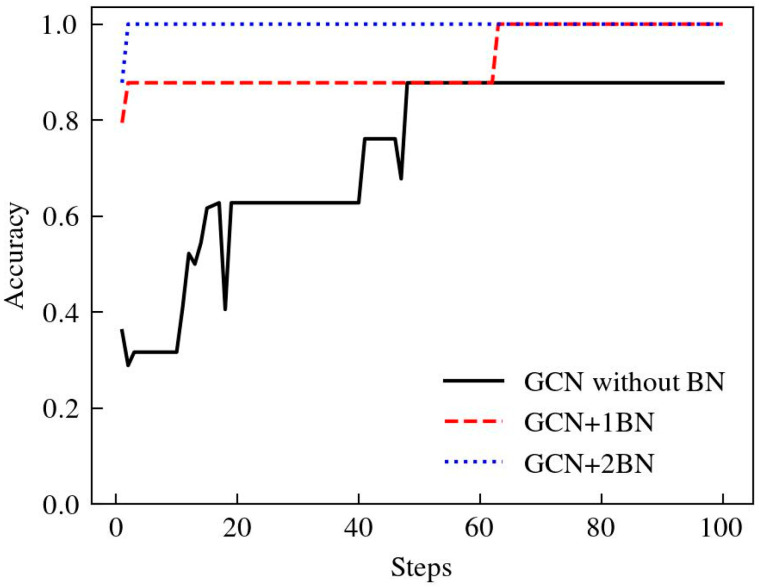
Accuracy of fault diagnosis with 0 layers, 1 layer, 2 layers of BN, respectively.

Batch normalization further standardized the time-domain information processing for each layer of the network output [42]. While improving the operational efficiency and generalization of the neural network, by adding the BN layer after two GCN networks, the network accuracy rate increased from 87.78% to 100%, each step of the training time decreased from 87 s to 9.17 s, and the algorithm convergence speed had been greatly improved as shown in Figure 9. Each step was to perform serial operations on seven graph data, and each graph was optimized for gradient descent after classification.

**Figure 10:**
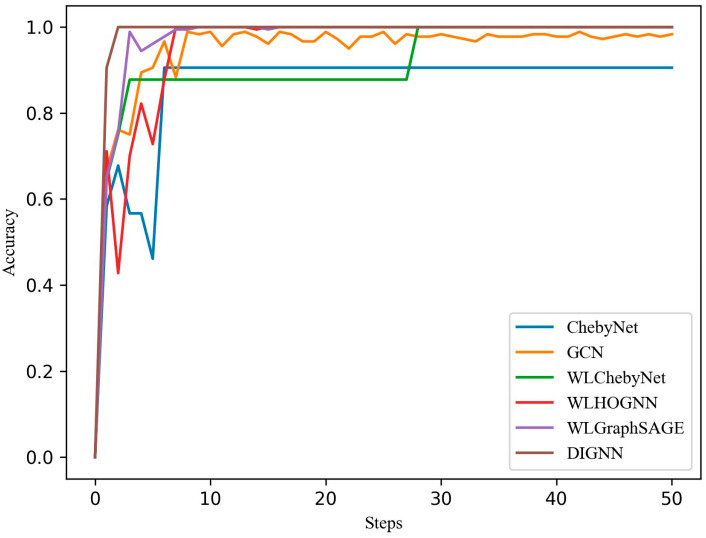
Comparison of coupling fault diagnosis accuracy between ChebyNet, GCN, WLChebyNet, WLGraphSAGE, WLHOGNN, and DIGNN.

Several models were used to diagnose coupling faults including ChebyNet, GCN, DVChebyNet, DVGraphSAGE, DVHOGNN, and the DIGNN proposed in the article. Among the models, except for ChebyNet and GCN, the models used a wavelet transform operation and consisted of a 100 × 1024 × 1024 CNN layer to convert the time–frequency domain data matrix into a one-dimension vector. These models used different graph neural network layers, and their network structure hyperparameters remained consistent. The accuracy of model diagnosis is shown in Figure 10. Due to the convolution operation on the input signal in the frequency domain, the ChebyNet had a faster convergence rate for the original data without wavelet transform. However, compared with the pre-processed model after wavelet transform, there was a large gap in accuracy. The GCN algorithm is a spatial domain graph neural network algorithm that has strong model fitting ability, but the time series data need to be iterated many times to discover the information contained in it. The graph neural network with wavelet transform can optimize the model input, avoid the uncertainty caused by pure data drive, and integrate its powerful model fitting ability to improve the accuracy of fault diagnosis. The DIGNN algorithm proposed in this paper had the highest accuracy in the validation set and showed great robustness in the test set. Additionally, it converged fastest among the benchmark models, as shown in Table 2.

**Figure 11:**
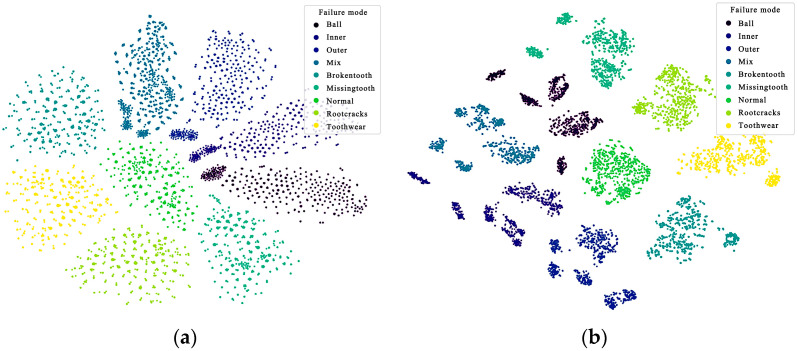
Visualization of the coupling fault characteristics. (**a**) Feature space after the first GCN layer; (**b**) feature space after the second GCN layer.

The graph neural network extracts the correlation information of the coupled fault components. Then, the graph dataset is input into the trained model, and the fault features are achieved from the output of the first GCN layer and the output of the second GCN layer, respectively. The visualization effect is shown in Figure 11. In the output of the first GCN layer, as shown in Figure 11a, compared with Figure 8, the normal state is at the coordinate zero of the feature space. However, the correlation between the coupling fault and its fault components is unclear. The boundary between the bearing fault and gear fault and the fault coupling mode are more clearly visualized in the second layer GCN output, as illustrated in Figure 11b.

**Figure 12:**
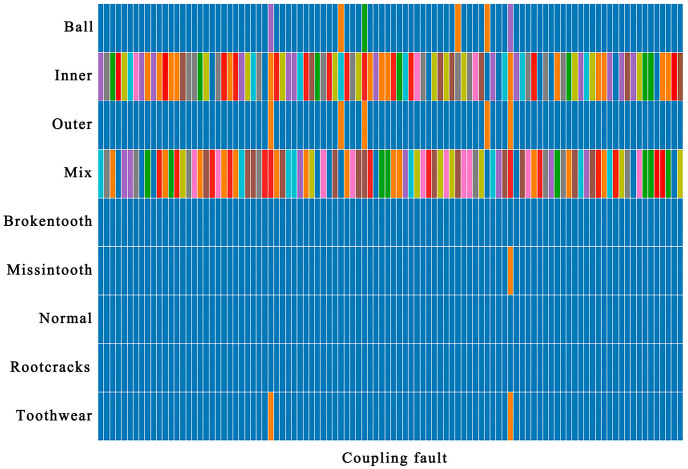
Main components of coupling faults based on the dynamic vertex output vector. Faults that may not be coupling factors are indicated in blue, and the three most possible coupling faults are highlighted in other colors.

For bearings with coupling faults, 100 coupling fault samples were selected, and their output after passing through the Softmax layer of the DIGNN network is shown in Figure 12. First of all, it ensures the accuracy of the coupling fault classification, and then, the fault coupling information is mined, a benefit from the advantages of graph neural network correlation analysis, and the fault coupling mode is revealed in the classification results. For each coupling fault sample, the blue part indicates that the coupling degree of this type of fault is low. Other colors indicate that the fault type is highly coupled. The coupling fault is mainly reflected in the inner ring fault, followed by the ball and the outer ring fault being reflected, and the coupling with the gear fault is better avoided.

Take the largest four items in the coupling fault classification vector $\hat{Y}$ among 100 fault coupling samples, and define the coupling fault diagnosis accuracy rate as $A_{n}=F_{n}/N_{X}$, where $F_{n}$ is the number of occurrences of each coupling fault, and $N_{X}$ is the number of samples (100 in this case). Then, except for the coupling fault $y_{3}$, the classification accuracy rate of the other three types of coupling components is shown in Table 3. The comprehensive diagnosis accuracy is defined as $\bar{A}=\sum A_{n}/n$, where n is the number of coupling fault components, and the comprehensive diagnosis accuracy of coupling fault in the dataset proposed in this paper was calculated at 88.3%, which can provide a reference for the analysis of coupling fault components.

## 5. Discussion and Conclusions

This paper presented a coupling fault diagnosis method based on a graph neural network. Through wavelet transform, a one-dimensional vibration signal was transformed into a time–frequency domain two-dimensional signal, which avoids the uncertainty caused by the data driven neural network. By establishing the fault coupling topology diagram, the coupling fault is diagnosed by using the graph neural network. The method proposed in this paper can achieve 100% accuracy of fault diagnosis under a single fault, and supports the mining of fault coupling information. The method proposed in this paper provides an interpretable data-driven method for fault coupling analysis, but the fault coupling dataset was limited, and more experiments are needed to verify the robustness of the proposed method.

## Figures and Tables

**Table 1:** Commonly used notations.

Notions	Descriptions
𝒢	Graph data
𝒱	Vertex set
ε	Edge set
A	Adjacent matrix
$\tilde{A}$	Adjacent matrix with self-loop
D	Degree matrix
${*}_{𝒢}$	Graph convolution operation
⊙	Hadamar product
L	Laplacian matrix
U	Fourier basis matrix of L
Λ	Eigenvalue matrix of L
λ	Eigenvalue forms Λ
X	Feature matrix of a graph
x, g	The feature vector of a graph
y	True label of x
$\hat{y}$	Predicted label of x
$g_{\theta }$	Filter parameterized by θ
T	Chebyshev polynomial coefficients
σ	Nonlinear activation function
θ, W	Learnable model parameters
ψ	Family of wavelets
α	Weight of the loss function Loss2
$A_{n}$	Diagnosis accuracy of coupled fault n
$\bar{A}$	Comprehensive diagnosis accuracy of coupling faults

**Table 2:** Comparison of the coupling fault diagnosis performance.

Models	Validation Accuracy	Test Accuracy	Steps to Convergence
ChebyNet	90.56%	82.78%	7
GCN	98.33%	98.89%	/
WLChebyNet	100%	96.67%	29
WLHOGNN	100%	99.45%	10
WLGraphSAGE	100%	97.8%	8
DIGNN	100%	100%	2

**Table 3:** Accuracy of the diagnosis of main components constituting coupling fault.

Fault Mode	Inner	Ball	Outer
1st obvious fault	100%	0	0
2nd obvious fault	0	94%	6%
3rd obvious fault	0	6%	59%
Coupling fault diagnosis accuracy	100%	100%	65%
Comprehensive accuracy	88.3%		

## Data Availability

Data is unavailable due to privacy.
